# Food Adulteration Law

**Published:** 1881-08

**Authors:** 


### THE PASSAGE OF THE FOOD ADULTERATION LAW.

[From the Sanitary Engineer.]

It is with no little pride and gratification that we are able to inform our readers that we have been at last successful in securing the passage in this State and New Jersey of the "Law to Prevent the Adulteration of Food and Drugs" which was prepared by the experts who were appointed by the National Board of Trade to award prizes in our food adulteration competition. For more than two years we have printed articles from the pens of Professor Henry Morton, C. F. Chandler, Prof. Wm. Ripley Nichols, and Messrs. S. P. Sharples, the late W. H. Merrick, G. W. Wigner and others, on the subject of the adulteration of food and drugs, and we became firmly convinced that, in dealing with this question, none but the most able, honest, and cautious men should be intrusted with the administration of the laws intended to check the evil.

On examination of the law, printed elsewhere, it will be seen that the whole matter is left largely to the discretion of the State Board of Health. Fortunately the Board is composed of men, who in our judgment, are equal to the duty imposed. The unanimity with which the most prominent wholesale grocers, druggists, and sugar refiners in the United States were willing to intrust this matter to the Board of Health shows a confidence in their capacity which is certainly complimentary.

Many questions will arise in the administration and enforcement of this law of the utmost importance to the commercial interests affected by it. As those interests have aided us in every way in securing the passage of the bill, we consider it our duty and shall make it our privilege to devote a department of this journal, to be in charge of a well known chemist and a well known pharmacist, to the discussion of these questions as they arise.

The people of this State are to be congratulated, and, if the law is efficiently enforced, as we expect it will be, the public health will be protected, and the food products and pharmaceutical preparations made and sold in this State will command a premium over those of States where similar laws do not exist or are not enforced.

We wish now to express our obligations to the press for their assistance, and especially to the *American Grocer*, *Druggist's Circular*, and *New Remedies;* and also to the New York *Sun*, *Nation*, and *Times*. We must not forget to name those gentlemen in the State Legislature who took an active interest in the passage of the bill: Speaker Sharpe, and Assemblymen Hon. Erastus Brooks, Wm. S. Andrews, Dr. Palmer, Chas. W. Dayton; to Senators Murtha and Astor; and to Dr. W. H. Newell, whose efforts very materially aided in securing the passage of the bills in both States. As might be expected, the medical profession took an active interest in it, and many members of it aided us greatly.

### The Food Adulteration Law.

[The following is the text of the law to prevent adulteration of food and drugs in this State, approved by the Governor, June 2.]

Section 1. No person shall, within this State, manufacture, have, offer for sale, or sell any article of food or drugs which is adulterated within the meaning of this Act, and any person violating this provision shall be deemed guilty of a misdemeanor, and, upon conviction thereof, shall be punished by fine not exceeding fifty dollars for the first offence, and not exceeding one hundred dollars for each subsequent offence.

Sec. 2. The term "food," as used in this Act, shall include every article used for food or drink by man. The term "drug," as used in this Act, shall include all medicines for internal or external use.

Sec. 3. An article shall be deemed to be adulterated within the meaning of this Act—

*a.* In the case of drugs.

1. If, when sold under or by a name recognized in the United States Pharmacopœia, it differs from the standard of strength, quality, or purity laid down therein.

2. If, when sold under or by a name not recognized in the United States Pharmacopœia, but which is found in some other pharmacopœia or other standard work on Materia Medica, it differs materially from the standard of strength, quality orpurity laid down in such work.

3. If its strength or purity fall below the professed standard under which it is sold.

*b.* In the case of food or drink.

1. If any substance or substances has or have been mixed with it so as to reduce or lower or injuriously affect its quality or strength.

2. If any inferior or cheaper substance or substances have been substituted wholly or in part for the article.

3. If any valuable constituent of the article has been wholly or in part abstracted.

4. If it be an imitation of or be sold under the name of another article.

5. If it consists wholly or in part of a deceased or decomposed, or putrid or rotten, animal or vegetable substance, whether manufactured or not, or in the case of milk, if it is the produce of a diseased animal.

6. If it be colored, or coated, or polished, or powdered, whereby damage is concealed, or it is made to appear better than it really is, or of greater value.

7. If it contain any added poisonous ingredient, or any ingredient which may render such article injurious to the health of a person consuming it : Provided, that the State board of health may, with the approval of the Governor, from time to time declare certain articles or preparations to be exempt from the provisions of this Act ; and provided further, that the provisions of this Act shall not apply to mixtures or compounds recognized as ordinary articles of food, provided that the same are not injurious to health and that the articles are distinctly labelled as a mixture, stating the components of the mixture.

SEC. 4. It shall be the duty of the State Board of Health to prepare and publish from time to time, lists of the articles, or compounds declared to be exempt from the provisions of this act in accordance with the preceding section. The State Board of Health shall also from time to time fix the limits of variability permissible in any article of food, or drug, or compound, the standard of which is not established by any national pharmacopœia.

SEC. 5. The State Board of Health shall take cognizance of the interests of the public health as it relates to the sale of food and drugs and the adulteration of the same, and make all necessary investigations and inquiries relating thereto. It shall also have the supervision of the appointment of public analysts and chemists, and upon its recommendation, whenever it shall deem any such officers incompetent, the appointment of any and every such officer shall be revoked and be held to be void and of no effect. Within thirty days after the passage of this Act, the State Board of Health shall meet and adopt such measures as may seem necessary to facilitate the enforcement of this Act, and prepare rules and regulations with regard to the proper methods of collecting and examining articles of food or drugs, and for the appointment of the necessary inspectors and analysts; and the State Board of Health shall be authorized to expend, in addition to all sums already appropriated for said board, an amount not exceeding ten thousand dollars, for the purpose of carrying out the provisions of this Act, and the sum of ten thousand dollars is hereby appropriated out of any money in the treasury not otherwise appropriated, for the purposes in this section provided.

SEC. 6. Every person selling or offering or exposing any article of food or drugs for sale, or delivering any article to purchasers, shall be bound to serve or supply any public analyst or other agent of the State or local board of health appointed under this Act, who shall apply to him for that purpose, and on his tendering the value of the same, with a sample sufficient for the purpose of analysis of any article which is included in this Act, and which is in the possession of the person selling, under a penalty not exceeding fifty dollars for the first offence, and one hundred dollars for a second and subsequent offences.

SEC. 7. Any violation of the provisions of this Act shall be treated and punished as a misdemeanor; and whoever shall impede, obstruct, hinder, or otherwise prevent any analyst, inspector, or prosecuting officer in the performance of his duty, shall be guilty of a misdemeanor, and shall be liable to indictment and punishment therefor.

SEC. 8. Any Act or parts of Acts inconsistent with the provisions of this Act are hereby repealed.

SEC. 9. All the regulations and declarations of the State Board of Health made under this Act, from time to time, and promulgated, shall be printed in the statutes at large.

SEC. 10. This Act shall take effect at the expiration of ninety days after it shall become a law.

### AN ACKNOWLEDGMENT.

The adulteration act proposed by the National Board of Trade has become a law in the States of New York and New Jersey; and we congratulate the public and the business community upon the auspicious beginning which has thus been made towards securing a sensible and practical regulation of the sale of food and drugs. The well-meant attempts heretofore made in this direction have largely failed, simply because sufficient study had not been given to the subject; and the absence of proper regulation gave opportunity for many sensational and misleading statements regarding an existing evil, to the injury of American manufactures in all parts of the globe. We are the more pleased that the present movement should have originated in New York, and that it has recived the intelligent and hearty support of her leading houses, both in the line of food and drugs. It should, and doubtless will, give increased confidence in the quality of goods supplied from this market ; and we hope soon to see a similar law enacted in every State, as well as by Congress, so that both State and inter-State commerce may experience the benefits which such a law unquestionably confers. It is but just to state that to Mr. Henry C. Meyer, the proprietor of *The Sanitary Engineer*, is due the credit of conceiving and, as chairman of the special committee appointed by the National Board of Trade, of carrying out the idea to its present degree of success. Neither is this the only good work which this paper has inaugurated ; the prize competition for plans for improved tenement-houses, in a sanitary point of view, having been set on foot and carried through to a successful termination by this enterprising journal, which has now become the leading authority in this country upon sanitary questions.—*The American Grocer.*

